# Functional Characterization of the *Xanthophyllomyces dendrorhous* Farnesyl Pyrophosphate Synthase and Geranylgeranyl Pyrophosphate Synthase Encoding Genes That Are Involved in the Synthesis of Isoprenoid Precursors

**DOI:** 10.1371/journal.pone.0096626

**Published:** 2014-05-05

**Authors:** Jennifer Alcaíno, Ignacio Romero, Mauricio Niklitschek, Dionisia Sepúlveda, María Cecilia Rojas, Marcelo Baeza, Víctor Cifuentes

**Affiliations:** 1 Departamento de Ciencias Ecológicas, Facultad de Ciencias, Universidad de Chile, Santiago, Chile; 2 Departamento de Química, Facultad de Ciencias, Universidad de Chile, Santiago, Chile; University Paris South, France

## Abstract

The yeast *Xanthophyllomyces dendrorhous* synthesizes the carotenoid astaxanthin, which has applications in biotechnology because of its antioxidant and pigmentation properties. However, wild-type strains produce too low amounts of carotenoids to be industrially competitive. Considering this background, it is indispensable to understand how the synthesis of astaxanthin is controlled and regulated in this yeast. In this work, the steps leading to the synthesis of the carotenoid precursor geranylgeranyl pyrophosphate (GGPP, C_20_) in *X. dendrorhous* from isopentenyl pyrophosphate (IPP, C_5_) and dimethylallyl pyrophosphate (DMAPP, C_5_) was characterized. Two prenyl transferase encoding genes, *FPS* and *crtE*, were expressed in *E. coli*. The enzymatic assays using recombinant *E. coli* protein extracts demonstrated that *FPS* and *crtE* encode a farnesyl pyrophosphate (FPP, C_15_) synthase and a GGPP-synthase, respectively. *X. dendrorhous* FPP-synthase produces geranyl pyrophosphate (GPP, C_10_) from IPP and DMAPP and FPP from IPP and GPP, while the *X. dendrorhous* GGPP-synthase utilizes only FPP and IPP as substrates to produce GGPP. Additionally, the *FPS* and *crtE* genes were over-expressed in *X. dendrorhous,* resulting in an increase of the total carotenoid production. Because the parental strain is diploid, the deletion of one of the alleles of these genes did not affect the total carotenoid production, but the composition was significantly altered. These results suggest that the over-expression of these genes might provoke a higher carbon flux towards carotenogenesis, most likely involving an earlier formation of a carotenogenic enzyme complex. Conversely, the lower carbon flux towards carotenogenesis in the deletion mutants might delay or lead to a partial formation of a carotenogenic enzyme complex, which could explain the accumulation of astaxanthin carotenoid precursors in these mutants. In conclusion, the *FPS* and the *crtE* genes represent good candidates to manipulate to favor carotenoid biosynthesis in *X. dendrorhous*.

## Introduction

Isoprenoids, also known as terpenoids, are one of the largest and most diverse families of natural products, consisting of over 40,000 structurally different compounds isolated from animals, plants and microorganisms [1]. Many biological functions have been attributed to them, including their roles as defensive and photoprotective agents, important components of cell membranes and both pigments and reproductive hormones, making them very attractive biotechnological metabolites [2]. These roles and structural diversity have attracted the attention of researchers from various fields of science to study isoprenoids function, biosynthesis and synthesis regulation.

**Figure 1:**
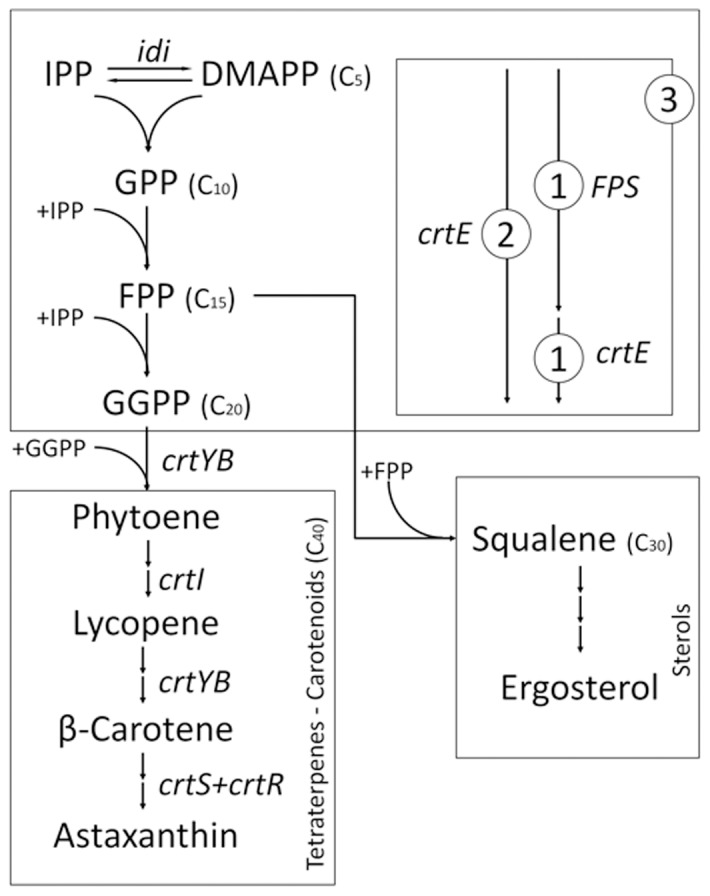
Synthesis of isoprenoids in *X. dendrorhous.* Metabolite abbreviations: IPP (isopentenyl pyrophosphate), DMAPP (dimethylallyl pyrophosphate), GPP (geranyl pyrophosphate), FPP (farnesyl pyrophosphate) and GGPP (geranylgeranyl pyrophosphate). The arrows represent the catalytic step with the respective enzyme-encoding gene. Genes controlling the steps shown in the figure are written in italics: *idi* [Genbank: DQ235686, IPP-isomerase], *crtE* [Genbank: DQ012943, GGPP-synthase], *FPS* [Genbank: KJ140284, FPP-synthase], *crtYB* [Genbank: DQ016503, Phytoene-β-carotene synthase), *crtI* [Genbank: Y15007, Phytoene desaturase], *crtS* [Genbank: EU713462, astaxanthin synthase] and *crtR* [Genbank: EU884133, Cytochrome P450 reductase]. The three proposed model systems to achieve the synthesis of GGPP are shown: 1) first system, a FPP- and a GGPP- synthase are involved and act sequentially, 2) second system, only one GGPP synthase is involved and 3) third system, participation of the first and second system simultaneously.

Despite the enormous structural diversity of isoprenoids, their biosynthesis shares the same initial steps. These steps correspond to the sequential union of a C_5_-isoprene unit by prenyl transferase enzymes [3]. The basic isoprene building blocks are the isopentenyl pyrophosphate (IPP) and its isomer dimethylallyl pyrophosphate (DMAPP). Generally, in eukaryotes and archaea, IPP derives from the mevalonate pathway [4], [5], while in most bacteria and plant plastids IPP is synthesized via the 2-C-methyl-D-erythritol 4-phosphate, DOXP/MEP, non-mevalonate pathway [6]. In the first step of isoprenoid biosynthesis (Fig. 1), IPP is converted into DMAPP, a reaction that is catalyzed by the isopentenyl pyrophosphate isomerase (encoded by *idi*). Then, a molecule of IPP is condensed with a molecule of DMAPP to form geranyl pyrophosphate (GPP; C_10_), the precursor of monoterpenes. The addition of a second IPP unit to GPP generates farnesyl pyrophosphate (FPP; C_15_), which is precursor of sesquiterpenes. The further addition of IPP to FPP results in geranylgeranyl pyrophosphate (GGPP; C_20_), the precursor of diterpenes. The later condensation of two molecules of GGPP yields phytoene, and that of two units of FPP gives squalene, which are the precursors of carotenoids and triterpenes (including sterols), respectively. In the synthesis of GGPP from DMAPP (DMAPP to GPP to FPP to GGPP), three different systems have been described, depending on the number of enzymes involved. In the first system, only one enzyme, a GGPP-synthase encoded by the *crtE* gene, produces GGPP from DMAPP [7]. In the second system, two enzymes are involved: a FPP-synthase (*FPS* gene) that forms FPP, followed by the GGPP-synthase to form GGPP [8]. The third system is a hybrid, where the first two systems act in parallel to give GGPP [9].

Carotenoids are natural pigments, fulfilling important physiological roles in a wide range of organisms. More than 750 chemical structures have been described to date [10], and plants, algae and some fungi and bacteria synthesize carotenoids. In general, animals are unable to biosynthesize carotenoids *de novo*, so they must obtain them through their diet [11]. Carotenoids are attractive from an industrial point of view because they are widely used as food colorants, antioxidants and nutraceutical agents [12]. Among the carotenoids, astaxanthin (3,3′-dihydroxy-β,β-carotene-4-4′-dione) stands out for its commercial potential, as it is currently being used as an antioxidant and as feed additive in aquaculture for salmon fish pigmentation [13], [14]. The biosynthesis of astaxanthin is limited to only a few organisms, including the basidiomycete yeast *Xanthophyllomyces dendrorhous* [15].

The astaxanthin biosynthesis in *X. dendrorhous* has been investigated, and the genes involved in the synthesis of astaxanthin from phytoene have been described. However, the early steps of carotenogenesis in this yeast are less known.

The *X. dendrorhous idi* [16] and *crtE* [17] genes have been described, but no *FPS* gene has been formally reported. This is interesting, as several metabolic engineering strategies have been attempted to increase carotenoid production in *X. dendrorhous*. To increase carotenoid precursors, the *idi* gene was over-expressed. However, that over-expression decreased the total amount of carotenoids [18]. Conversely, when the *crtE* gene was over-expressed, there was only a slight increase in the total carotenoid content [19]. An explanation for this result could be that the GGPP-synthase activity in *X. dendrorhous* is limited by the amount of FPP, if this is its only substrate.

In this work, we report the isolation of the *FPS* gene from *X. dendrorhous*. Through functional studies, we conclude that *FPS* is involved in the synthesis of FPP and that its gene product uses IPP and DMAPP, or IPP and GPP, as substrates, while the GGPP-synthase utilizes exclusively IPP and FPP as substrates to produce GGPP.

## Materials and Methods

### Microorganisms, growth conditions and enzymes

All strains used and/or created in this work are listed in Table 1. The wild-type UCD 67–385 (ATCC 24230) *X. dendrorhous* strain was used for the genomic and cDNA library construction [17] and as the parental strain for genetic modifications. The yeast strains were grown at 22°C with constant agitation in YM rich medium (1% glucose, 0.3% yeast extract, 0.3% malt extract and 0.5% peptone). Yeast transformants were selected on YM-agar plates (1.5% agar) that were supplemented with 12.5 µg/mL hygromycin-B.

**Table 1:** Strains used and/or constructed in this work.

Strains	Genotype or Relevant Features	Reference or Source
*E. coli*		
DH-5alpha	F- φ80d lacZΔM15Δ (lacZY-argF) U169 deoR recA1 endA1 hsdR17(rk- mk+) phoA supE44l- thi-1 gyrA96 relA1	[20]
BL21-Gold (DE3)	B F^-^ ompT hsdS(r_B_^−^m_B_^−^) dcm^+^ Tet^r^ gal λ(DE3) endA Hte	Stratagene
BL21+pET28	BL21-Gold (DE3) strain bearing plasmid pET28.	This work
BL21+FPS	BL21-Gold (DE3) strain bearing plasmid pET28-*FPS.*	This work
BL21+crtE	BL21-Gold (DE3) strain bearing plasmid pET28-*crtE.*	This work
*X. dendrorhous*		
UCD 67–385	ATCC 24230, wild-type. Diploid strain [42].	ATCC
385-FPS^(+/−)^	(*FPS* ^+^/*FPS^::hph^*). Heterozygote transformant derived from UCD 67–385 with a portion of one of the *FPS* alleles replaced by a hygromycin B resistance cassette.	This work
385-crtE^(+/−)^	(*crtE* ^+^/*crtE^::hph^*). Heterozygote transformant derived from UCD 67–385 with a portion of one of the *crtE* alleles replaced by a hygromycin B resistance cassette.	This work
385-XdVexp2^(+/−)^	(*int* ^+^/*int^::hph^*). Heterozygote transformant derived from UCD 67–385 with a non-coding genomic region (*locus int*) replaced by an empty over-expressing cassette (without an inserted ORF) and a hygromycin B resistance cassette.	This work
385-FPS^(+/+, +1)^	(*int* ^+^/*int^::hph+FPS^*). Transformant derived from UCD 67–385 containing an additional *FPS* allele and a hygromycin B resistance cassette integrated at *locus int*.	This work
385-crtE^(+/+, +1)^	(*int* ^+^/*int^::hph+crtE^*). Transformant derived from UCD 67–385 containing an additional *crtE* allele and a hygromycin B resistance cassette integrated at *locus int*.	This work

ATCC: American Type Culture Collection.

*Locus int* [Genbank: KJ140286].

*E. coli* DH-5alpha was used for plasmid propagation and was grown at 37°C in Luria- Bertani (LB) medium, supplemented with 100 µg/mL ampicillin for plasmid selection and 20 µg/mL of X-gal (5-bromo-4chloro-3-indolyl-β-D-galactopyranoside) for recombinant clone selection by blue-white screening [20]. When necessary, recombinant clones were selected by direct colony PCR with a comprehensive set of primers. For the *E. coli* heterologous expression of the *FPS* and *crtE* genes, the BL21 strain was used and grown at 37°C in LB medium supplemented with 50 µg/mL kanamycin.

*Taq* DNA polymerase (pol), restriction enzymes, Klenow polymerase and M-MLV reverse transcriptase were purchased from Promega, and the *Pfu* DNA polymerase was purchased from Invitrogen.

### Nucleic acids extraction

DNA extraction was performed from protoplasts of *X. dendrorhous* according to [21], and total RNA extraction was performed according to a modified protocol of Chomczynski and Sacchi [22], [23]. Briefly, cell pellets were suspended in 200 µL of lysis buffer (0.5 M Sodium acetate pH 5.5, 5% SDS, 1 mM EDTA) and broken through mechanical rupture with 0.5 mm glass beads (BioSpec) and shaking in a mini bead beater-16 (BioSpec) for 1 min. Then, 800 µL of Tri-Reagent (Ambion) was added, followed by shaking in the bead beater for 1 min and incubation for 10 min at room temperature. Then, 200 µL of chloroform was added, followed by mixing and incubation for 6 min and centrifugation for 10 min at 14,000×g. RNA was extracted from the recovered aqueous phase by precipitation with 1 volume of isopropanol and 0.5 volume of precipitation buffer (1.2 M NaCl, 0.8 M sodium citrate) for 1 h at −20°C. The RNA was washed with 70% ethanol, suspended in RNase-free H_2_O and quantified spectrophotometrically at 260 nm according to [20] in a V-630 UV-Vis Spectrophotometer from JASCO.

### DNA amplification and sequence analyses

The oligonucleotides that were designed and used in this study were synthesized by Integrated DNA Technologies and are listed in Table S1. The DNA amplification reactions were performed in a final volume of 25 µL containing 2 U of *Taq* DNA pol, 2.5 µL of 10X *Taq* buffer, 0.5 µL of 10 mM dNTPs, 1 µL of 50 mM MgCl_2_, 1 µL of 25 µM of each primer and 10–20 ng of template DNA. In general, the PCR reactions were performed in a 2720 Applied Biosystems thermal cycler with the following program: initial denaturation at 95°C for 3 min; 35 cycles of denaturation at 94°C for 30 s, annealing at 55°C for 30 s, synthesis at 72°C for 3 min and a final extension step at 72°C for 10 min. The samples were kept at 4°C until analyzed by 0.8% agarose gel electrophoresis in TAE buffer containing 0.5 µg/mL ethidium bromide [20]. DNA for sequencing or plasmid construction was purified from gels using the glass milk method [24]. The nucleotide sequences were obtained from an ABI 3100 Avant genetic analyzer using the BigDye terminator v3.1 kit (Applied Biosystems). DNA sequences were analyzed using Geneious 6.0.4, CLUSTAL W 1.8 and programs that are available at the NCBI web site.

### Single-strand DNA synthesis and quantitative RT-PCR (RT-qPCR)

The cDNA was synthesized using M-MLV reverse transcriptase (Invitrogen) with 5 µg of total RNA in a final volume of 20 µL, according to the manufacturer's protocol. The relative transcript level analyses for each gene were performed in an Mx3000P quantitative PCR system (Stratagene) using 1 µL of the reverse transcription reaction, 0.25 µM of each primer (Table S1) and 10 µL of the SensiMix SYBR Green I (Quantace) kit in a final volume of 20 µL. The pairs of primers used had efficiencies greater than 95%, as determined by standard curves with a correlation coefficient of R2≥0.996. The Ct values obtained were normalized to the respective value of the *X. dendrorhous* actin gene [Genbank: X89898.1] [25] and were later expressed as a function of the control conditions using the ΔΔCt algorithm [26].

### Obtaining the genes controlling the GGPP synthesis from IPP in *X. dendrorhous*

According to the Kyoto Encyclopedia of Genes and Genomes [27], three enzymatic activities, EC 2.5.1.1 (dimethylallyl transtransferase), EC 2.5.1.10 (geranyl transtransferase) and EC 2.5.1.29 (GGPP-synthase) are involved in the synthesis of GGPP from IPP and DMAPP. In *S. cerevisiae,* the dimethylallyl- and the geranyl-transtransferase activities are performed by the FPP-synthase encoded by the *ERG20* gene [28], and the *BTS1* gene, which is homologous to the *crtE* gene described in other organisms, encodes the GGPP-synthase activity [29]. Only the *X. dendrorhous crtE* gene has been previously described [17]. To find the *X. dendrorhous FPS* gene, a BLASTp analysis was performed using the deduced amino acid sequences from *ERG20* as a query. By this way, the homologous genes from the basidiomycetes *Coprinopis cinnerea, Cryptococcus neoformans*, *Laccaria bicolor* and *Ustilago maydis* were obtained [Genbank: EAU81564.1, XP_571137, DS547151 and XP_757593, respectively], and the deduced amino acid sequences were aligned. From the conserved regions, primers were designed to amplify by PCR the *X. dendrorhous FPS* gene. By this way, a *X. dendrorhous* genomic PCR amplification product of approximately 800 bp (using primers FPSF and FPSR) was obtained, which was completely sequenced. BLAST analyses of the obtained nucleotide sequence against the patent database available at the National Center of Biotechnology Information (NCBI), showed 100% identity with the *X. dendrorhous FPS* gene sequence from patent EP0955 363A2 [30]. This patent described the *X. dendrorhous FPS* gene isolation and reported its sequence [Genbank: AR349868.1], but it did not analyzed the gene functionality nor its impact on the yeast carotenogenesis. Nevertheless, primers were designed from this sequence to amplify the *X. dendrorhous FPS* gene.

### Plasmid construction for E. coli and *X. dendrorhous* transformations

All plasmids used and constructed in this work are listed in Table 2.

**Table 2:** Plasmids used and/or constructed in this work.

Plasmid	Genotype or Relevant Features	Source or Reference
pBluescript SK- (pBS)	ColE1 ori; AmpR; cloning vector with blue-white selection	Stratagene
pMN-*hph*	pBS bearing the hygromycin B (*hph*) resistance cassette at the *Eco*RV site.	[17]
pXd-g*crtE*	pBS containing a 3.3 kb DNA fragment carrying the *crtE* gene of *X. dendrorhous*.	[17]
pXd-g*crtE*::*hph*	pXD-*crtE* with a 1,984 bp *Eco*RV fragment deletion of the *crtE* gene of *X. dendrorhous* and replaced by the *hph* cassette from pMN-*hph*.	[17]
pXd-g*FPS*	pBS containing a 2.5 kb DNA fragment carrying the *FPS* gene of *X. dendrorhous*.	This work
pXd-g*FPS*::*hph*	pXD-FPS with a 2,038 bp *Eco*RV-*Bgl*ll fragment deletion of the *FPS* gene of *X. dendrorhous* and replaced by the *hph* cassette from pMN-*hph*.	This work
pXd-c*crtE*	pBS containing the cDNA version of the *crtE* gene from *X. dendrorhous*	This work
pXd-c*FPS*	pBS containing the cDNA version of the *FPS* gene from *X. dendrorhous*	This work
pXdVexp2	*X. dendrorhous* expression vector: pBS bearing the *X. dendrorhous* ubiquitin promoter [Genbank: KJ140285] and GPD terminator [Genbank:Y08366] with a *Bam*HI site between them to insert the gene to express and the hygromycin B cassette for selection, flanked by non-coding genomic [Genbank: KJ140286] regions to target the construction integration in the genome.	This work
pXdVexp2-c*crtE*	pXdVexp2 bearing the cDNA version of the *crtE* gene from *X. dendrorhous.*	This work
pXdVexp2 -c*FPS*	pXdVexp2 bearing the cDNA version of the *FPS* gene from *X. dendrorhous*.	This work
pET28a(+)	*E. coli* expression vector. pBR322 ori; KanR; LacI repressor.	Novagen
pET28-c*crtE*	pET28a(+) bearing the cDNA version of the *X. dendrorhous crtE* gene.	This work
pET28-c*FPS*	pET28a(+) bearing the cDNA version of the *X. dendrorhous FPS* gene.	This work

A 3,312 bp *Eco*RI/*Dra*I DNA fragment containing the *X. dendrorhous crtE* gene was sub-cloned from plasmid pXD-I10 [17] into the pBluescript SK- vector, giving rise to pXd-g*crtE*. The plasmid pXd-g*FPS* was constructed by inserting a 2,627 bp PCR-amplified DNA fragment encoding the *FPS* gene into the *Eco*RV site of the pBluescript SK-. This last DNA fragment was amplified using primers FPSORF1F and FPSORF1R (Table S1) and genomic DNA of the UCD 67–385 wild-type strain as template. The genomic versions of both genes from the wild-type strain UCD 67–385 were uploaded at the Genbank database as follows: *crtE* [Genbank: DQ012943.1] and *FPS* [Genbank: KJ140284].

Next, from the genomic *crtE* and *FPS* gene versions, primers were designed to amplify their cDNA obtained by reverse transcription (RT) of total RNA from the wild-type strain UCD 67–385. The amplified products were also cloned into the pBluescript SK- vector, resulting in the plasmids pXd-c*crtE* and pXd-c*FPS*, whose inserts were completely sequenced. The plasmids pET28-c*crtE* and pET28-c*FPS* for heterologous expression in *E. coli* were constructed by PCR amplification of the cDNA of each gene from plasmids pXd-c*crtE* and pXd-c*FPS*, respectively, and using primers with restriction sites (Table S1) for an in-frame ligation into the *Nco*I+*Nde*I digested plasmid pET28a.

The plasmids pXd-g*crtE*::*hph* and pXd-g*FPS*::*hph* were constructed as shown in Figure S1. For pXd-g*crtE*::*hph,* a 1,984 bp *Eco*RV DNA fragment in pXD-gcrtE was replaced by the hygromycin B resistance cassette obtained from the digestion of pMN-*hph* with *Eco*RV; in the case of pXd-g*FPS*::*hph*, a 2,038 bp *Eco*RV/*Bgl*II DNA fragment in pXd-gFPS was replaced by the hygromycin B cassette.

To over-express the *crtE* and *FPS* genes in *X. dendrorhous,* plasmids pXdVexp2-c*crtE* and pXdVexp2-c*FPS* were constructed as shown in Figure S1. Briefly, the *crtE* and the *FPS* cDNAs were amplified from plasmids pXd-c*crtE* and pXd-c*FPS*, respectively, and were then independently inserted at the *Hpa*I site of pXdVexp2. In this approach, after transformation, the gene to be over-expressed is integrated into the yeast genome at *locus int* [Genbank: KJ140286], which represents a non-coding DNA region that does not reveal an evident new phenotype when it is interrupted. Each cDNA is regulated by the promoter of the ubiquitin gene [Genbank: KJ140285] and the terminator from the glyceraldehyde-3-phosphate dehydrogenase [Genbank: Y08366] gene of *X. dendrorhous*.

For transformant DNA preparation, plasmids pXd-g*crtE*::*hph*, pXd-g*FPS*::*hph*, pXdVexp2-c*FPS* and pXdVexp2-c*crtE*, were linearized by digestion with *Kpn*I+*Sma*I, *Ava*I, *Not*I and *Not*I, respectively (Fig. S1).

### Transformation of *X. dendrorhous*

*X. dendrorhous* was transformed by electroporation [31] using a BioRad gene pulser X cell with PC and CE cassettes under the following conditions: 125 mF, 600 Ω and 0.45 kV [32]. The electrocompetent cells were prepared from an exponential culture at OD_600nm_ = 1.2, grown in YM medium and transformed with 1 to 5 µg of linear donor DNA. The resulting transformant strains were confirmed as *X. dendrorhous* through an analysis of the ITS1, 5.8 rRNA gene and ITS2 DNA sequences [33].

### Carotenoid and sterol extraction and RP-HPLC analyses

The acetone extraction method [34] was used for carotenoid extraction from cellular pellets. Total carotenoids were quantified spectrophotometrically at 465 nm using an absorption coefficient of A1% = 2,100 and normalized to the dry weight of the yeast. The extracted carotenoids were separated by RP-HPLC using a RP-18 Lichrocart125-4 (Merck) column with acetonitrile:methanol:isopropanol (85∶10∶5, v/v) as the mobile phase, with a 1 mL/min flux under isocratic conditions, and the elution spectra were recovered using a diode array detector. Carotenoids were identified according to their spectra and retention time in comparison to standards.

Sterols were extracted according to [35]. For this extraction, cell pellets were mixed with 4 g of KOH and 16 mL of 60% (v/v) ethanol/water and incubated at 80±2°C for 2 h. Non-saponificable sterols were extracted with 10 mL of petroleum ether, quantified spectrophotometrically at 280 nm using an absorption coefficient of A1% = 11.500 and normalized to the dry weight of the yeast. Sterols were separated by RP-HPLC with a C-18 column, using methanol:water (97∶3, v/v) as the mobile phase at 1 mL/min under isocratic conditions. The elution spectra were recovered using a diode array detector, and sterols were visualized in the 280 nm channel and compared to ergosterol standard purchased from Sigma-Aldrich.

### Protein extraction

Soluble proteins from *E. coli* were obtained by sonication of cell pellets (5 mL from a culture with a DO_600_ 0.5–0.7) suspended in 1 mL of extraction buffer, 50 mM MOPS pH 7.5 and protease inhibitor cocktail (Complete, Roche Biomedicals), using a Cole Parmer 4710 ultrasonic homogenizer. Twelve sonication pulses of 10 s at 40% duty, followed by 30 s of incubation in ice, showed the best protein yield from the soluble fraction. The lysate was centrifuged at 14,000×g, and the supernatant was recovered and stored at −80°C until its use.

Soluble proteins from *X. dendrorhous* were also obtained by sonication of cell pellets from 10 mL of a stationary phase culture. Each pellet was suspended in 5 mL of extraction buffer (50 mM MOPS, 5 mM EDTA, 0.5 M sorbitol and protease inhibitor cocktail) and ruptured by 15 pulses of sonication of 30 s at 90% duty followed by 1.5 min of incubation in ice. The lysate was sequentially centrifuged at 4,000×g for 5 min, 10,000×g for 20 min and 100,000×g for 1 h, recovering the supernatant from each step. The recovered supernatants were purified from endogenous metabolites through a PD-10 exclusion column (GE Healthcare). The recovered proteins were stored at −80°C.

### Prenyl transferase activity assays

Prenyl transferase activity assays were adapted from [36]. Two protein extract samples were obtained from two replicate cultures of each of the *X. dendrorhous* or *E. coli* strains that were assayed. The enzymatic assays were performed in duplicates in a final volume of 500 µL containing 12 µg of protein extract (14,000×g or 100,000×g supernatants), 50 mM MOPS, 5 mM MgCl_2_, 6 mM DTT, 100,000 dpm ^14^C-IPP (56.6 mCi/mmol; 0.81 nmol), 20.2 µM IPP and saturating concentrations of alternative allylic substrates (20.2 µM DMAPP, 16.4 µM GPP or 13.8 µM FPP). Incubations were performed at 22°C for 1 h. ^14^C-GPP, ^14^C-FPP and/or ^14^C-GGPP were expected as products, as was ^14^C-DMAPP possibly formed by an IPP isomerase present in the protein extracts. The corresponding ^14^C-prenyl alcohols, which are formed by phosphatase activities on the prenyl transferase products, may also be obtained, though the amount produced in most cases was negligible and thus not considered. Reactions were stopped by adding 500 µL of hexane to extract the ^14^C-prenyl alcohol phosphatase products. After extraction, the organic phase was separated and removed. The remaining aqueous phase was acidified with HCl to a final concentration of 0.66 N and incubated for 1 h at 37°C to hydrolyze allylic ^14^C-prenyl phosphates formed by prenyl transferases. The resulting labeled, rearranged prenyl alcohols were extracted with 500 µL of hexane and the organic phase (allylic hexane phase) was stored at −20°C for quantification and further thin layer chromatography (TLC) analysis. One enzyme unit (U) was defined as 1 µmol of product formed per min under the assay conditions. Prenyl transferase specific activity was expressed in U/mg of protein. A representative chromatogram prenyl product analysis by TLC is shown in Figure S2.

### Product quantification and analysis

^14^C-labelled products, as well as the remaining substrate, were quantified in the organic and aqueous phases, respectively, by liquid scintillation counting in a Perkin Elmer Tri-Carb 2810TR instrument. ^14^C-Prenyl alcohols in the allylic hexane phase (C_5_, C_10_, C_15_ and/or C_20_), which are formed from allylic prenyl phosphate products, were analyzed by TLC in 60 mm silica gel plates (TLC Silica gel 60 RP-18, Merck) developed with acetone/H_2_O (9∶1, v/v) as mobile phase. Standard dimethyl allyl alcohol (C_5_), linalool (C_10_), trans-nerolidol (C_15_) and geranylgeraniol (C_20_) (4 µg, 8 µg, 2 µg and 0.5 µg, respectively) were added to the samples that were concentrated under N_2_ up to 10–15 µL and loaded into the plates. The plates were sprayed with Libermann-Burchard solution (H_2_SO_4_:acetic anhydride:ethanol (1∶1∶8, v/v) and heated gently on a heating plate to reveal the standards (Rf values were 0.9, 0.8, 0.71 or 0.62 for the C_5_, C_10_, C_15_ or C_20_ standards, respectively). Segments of 2.5 mm were cut from the origin to the solvent front, and radioactivity was quantified in each fraction by liquid scintillation counting.

## Results and Discussion

### Sequence analysis of genes controlling the GGPP synthesis from IPP in *X. dendrorhous*

The *X. dendrorhous crtE* gene has been previously isolated and described [17], which encodes a 376 amino acid protein with a predicted molecular weight of 42.16 kDa. The *FPS* gene has 9 exons (of 135, 144, 81, 123, 89, 73, 141, 164, 118 pb) and 8 introns (of 189, 151, 122, 96, 113, 85, 97, 132 pb). The *FPS* gene encodes a 355 amino acid protein with a predicted molecular weight of 40.24 kDa.

**Figure 2:**
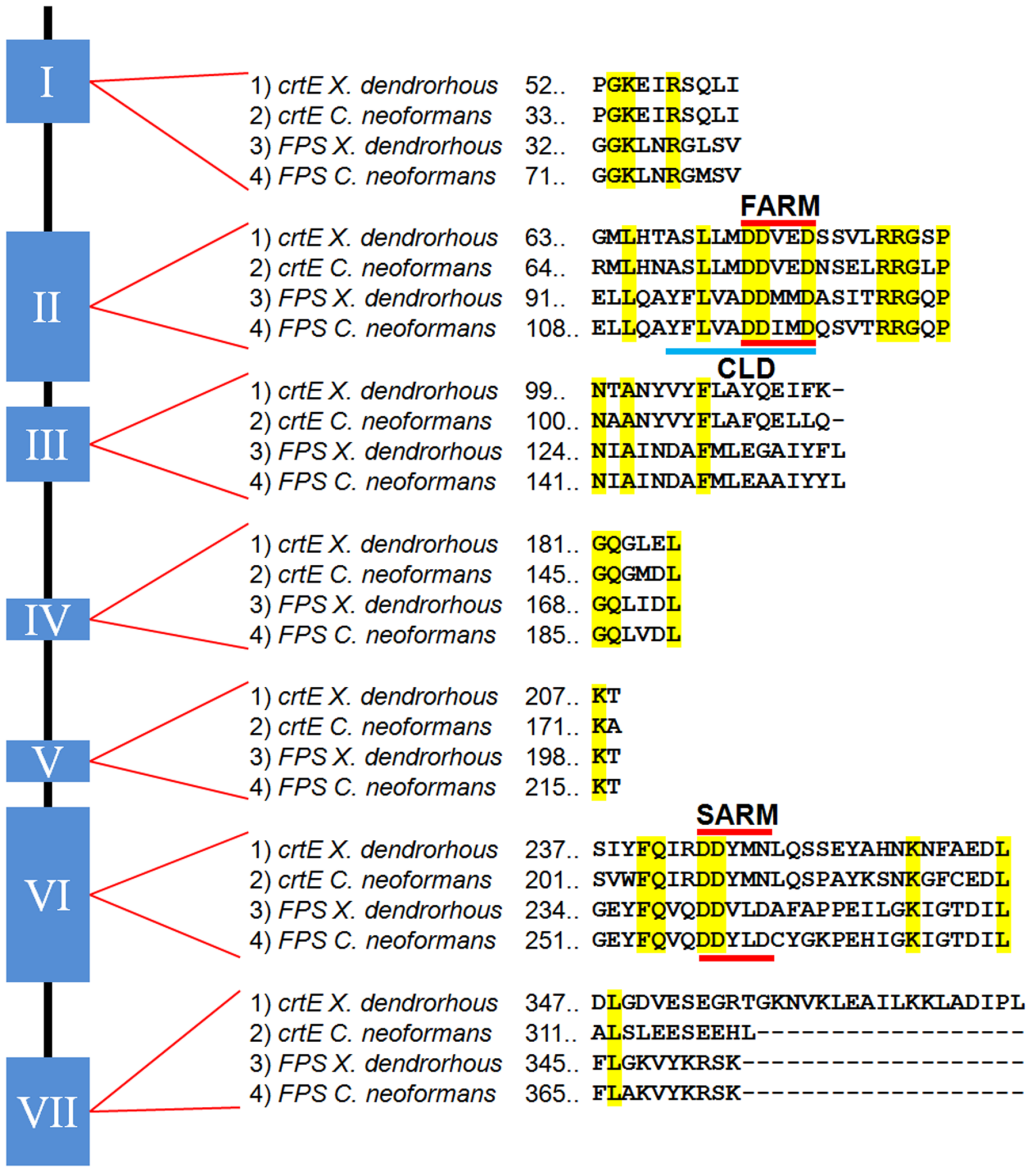
*X. dendrorhous* and *C. neoformans FPS* and *crtE* deduced amino acid sequence analysis. Seven conserved regions have been previously reported in prenyl transferase enzymes; among these are the two aspartic acid-rich motifs, FARM and SARM, and the CLD *chain length domain*, which is responsible for determining the resulting isoprene chain length. The sequence alignment shows similarity between the compared proteins, particularly for the aforementioned motifs. However, the seventh reported conserved region shows little or no agreement between the *crtE* and the *FPS* translated sequences. This was consistent for geranylgeranyl pyrophosphate synthases from other related organisms. Amino acid sequences were deduced from: *X. dendrorhous FPS* [Genbank: KJ140284] and *crtE* [Genbank: DQ012943.1]; *C. neoformans FPS* [Genbank: XP_571137] and *crtE* [Genbank: XP_572774].

Because the *crtE* and the *FPS* genes encode prenyl transferase enzymes, their deduced amino acid sequences were compared, including the amino acid sequences from *C. neoformans* (*crtE* [Genbank: XP_572774.1] and *FPS* [Genbank: XP_571137.1]) to increase the robustness of the analysis (Fig. 2). The amino acid identity between the homologous enzymes was 47% and 63% for the *crtE* and *FPS* gene products, respectively. As expected, the identity among the four enzymes decreased dramatically. Even though the sequence identity among the prenyl transferase enzymes encoded by the analyzed *crtE* and *FPS* genes is low, the previously reported conserved regions in these enzymes [37], [38] could be distinguished (Fig. 2). This was the case for the two-aspartic acid-rich motifs (DDxxD) that were recognized: i) the First Aspartic acid-Rich Motif (FARM; DDxxDxxxxRRG), and ii) the Second Aspartic acid-Rich Motif (SARM; GxxFQxxDDxxD). Moreover, the chain length domain (CLD) proposed by Ohnuma and co-workers [39] could be distinguished in the GGPP- and FPP-synthase deduced sequences. The *X. dendrorhous* GGPP-synthase contains a small amino acid and a serine at the fourth and fifth positions before the FARM motif, while FPP-synthase has a tyrosine and phenylalanine at these positions, which is consistent with the chain length determination of these two types of prenyl transferases [40], [41]. Although the predicted *X. dendrorhous* FPP-synthase shows the seven conserved regions (I to VII) described for this type of enzyme, the predicted GGPP-synthase from *X. dendrorhous* lacks any distinctive aminoacids from this last conserved region.

### Heterologous expression of FPS and crtE genes from *X. dendrorhous* in *E. coli*

To study the enzymatic activities encoded by the *FPS* and *crtE* genes from *X. dendrorhous*, the genes were independently expressed in *E. coli*. The synthesis of FPP-synthase and GGPP-synthase in the transformant strains (BL21+FPS and BL21+crtE) was confirmed by SDS-PAGE analyses (Fig. S3), which showed protein bands of the expected size (40.24 and 42.16 kDa, respectively) only when cultures were induced by IPTG. Moreover, the protein bands were excised from gels and analyzed by MALDI-TOF to confirm the identity of the proteins. Prenyl transferase assays were performed with protein extracts from transformant strains BL21+pET28, BL21+FPS and BL21+crtE (Table 1), using induced and non-induced cultures as controls. The results are summarized in Table 3. Prenyl transferase activity was not detected with any of the substrates in the extracts of non-induced cultures or in extracts from BL21+pET28. In contrast, ^14^C-labeled products were found in the allylic fraction from incubations with induced BL21+FPS and BL21+crtE extracts. The extracts from BL21+FPS formed C_10_ and C_15_^14^C-allylic pyrophosphates from DMAPP plus ^14^C-IPP, as evidenced by the C_10_ and C_15_^14^C-alcohols detected in the allylic hexanic phase (Table 3), while GPP was converted by BL21+FPS extracts into a C_15_ allyl pyrophosphate that was detected as a C_15_ alcohol after acid hydrolysis. Interestingly, no products were found in the allylic fraction when FPP plus ^14^C-IPP were utilized as substrates. In contrast, the extracts from BL21+*crtE* did not utilize DMAPP or GPP as substrates but were able to convert FPP in the presence of ^14^C-IPP to give C_20_^14^C-alcohols in the allylic fraction, which is evidence of ^14^C-GGPP synthesis. These results demonstrate that the prenyl transferases encoded by *FPS* or *crtE* differ strongly in their substrate specificity, in agreement with their sequence homology to the FPP- and GGPP-synthase encoding genes from other organisms. Thus, GGPP production for astaxanthin biosynthesis by *X. dendrorhous* would result from the combined action of both prenyl transferases, i.e., the FPP- and GGPP-synthases, with the latter providing a link to the central metabolism essential for astaxanthin production. This was demonstrated by the conversion of DMAPP plus ^14^C-IPP into C_20_^14^C-products by the combined BL21+*FPS* and BL21+*crtE* protein extracts, in addition to the C_15_ and C_10_ labeled products found in the hexane phase after acid hydrolysis (Table 3). However, the C_20_ labeled products fraction was lower than the other fractions, which would result from a low geranylgeranyl-pyrophosphate synthase (*crtE* gene) activity compared to farnesyl- pyrophosphate synthase (*FPS* gene) activity in the protein extracts from *E. coli* (Table 3). Thus, FPP accumulates and is only partially converted into GGPP. Product distribution in *E. coli* combined extracts is consistent with results found in the *X. dendrorhous* enzymatic assays with DMAPP as substrate in which 86% of the product corresponded to FPP and only 12% to GGPP (see below, Table 4). The geranylgeranyl-pyrophosphate synthase activity in *X. dendrorhous* is much lower than the farnesyl- pyrophosphate synthase activity (Table 4), thus FPP conversion to GGPP is a slow reaction.

**Table 3:** Prenyl transferase enzymatic assays with recombinant *E. coli* protein extracts.

Strain Protein Extract	Variable Substrate	Prenyl Transferase Activity (U/mg×10^6^)	Percentage of each Product*
BL21pET28	DMAPP	-	ND
	GPP	-	ND
	FPP	-	ND
BL21+FPS	DMAPP	6.06±0.75	C_10_ (12.9%), C_15_ (87.1%)
	GPP	3.84±0.43	C_15_ (100%)
	FPP	-	ND
BL21+crtE	DMAPP	-	ND
	GPP	-	ND
	FPP	2.44±0.16	C_20_ (100%)
BL21+FPS and BL21+crtE	DMAPP	6.65±0.11	C_10_ (17.4%), C_15_ (71.5%), C_20_ (11.1%)

Enzymatic assays were performed as described in the Materials and Methods section. Two protein extract samples from two replicate cultures were obtained for each strain, and then two enzymatic assays were performed with each protein extract. The table values are the mean values of prenyl transferase activity and percentage of product ± standard error of the mean.

*Percentage of each product considering 100% the total radioactivity found in the hexanic phase.

ND: Product not detected. C_10_, C_15_ and C_20_ correspond to products containing 10, 15 and 20 carbon atoms, respectively.

**Table 4:** Prenyl transferase enzymatic assays with *X. dendrorhous* protein extracts.

Strain Protein Extract	Variable Substrate	Prenyl Transferase Activity (U/mg×10^6^)	Percentage of each Product*		
			C_10_	C_15_	C_20_
UCD 67–385	DMAPP	5.0±0.15	2±0	86±4	12±5
	FPP**	1.15±0.35	ND	ND	ND
385-FPS^(+/+, +1)^	DMAPP	9.24±0.45	2±1	85±3	13±3
	FPP**	1.13±0.68	ND	ND	ND
385-crtE^(+/+, +1)^	DMAPP	4.54±0.76	3±1	84±4	13±4
	FPP	2.81±0.81	15±4	74±5	11±4
385-FPS^(+/−)^	DMAPP	3.79±0.6	2±1	86±3	12±4
	FPP**	0.64±0.15	ND	ND	ND
385-crtE^(+/−)^	DMAPP	4.1±1.06	3±1	84±2	14±3
	FPP**	0.74±0.03	ND	ND	ND

Enzymatic assays were performed as described in the Materials and Methods Section. Two protein extract samples from two replicate cultures were obtained for each strain, and then two enzymatic assays were performed with each protein extract. The table values are the mean values of prenyl transferase activity and percentage of product ± standard error of the mean.

*Percentage of each product considering 100% the total radioactivity found in the hexanic phase.

ND: Product not detected in TLC analysis. C_10_, C_15_ and C_20_ correspond to products containing 10, 15 and 20 carbon atoms, respectively.

**The assays conducted using FPP as a substrate yielded low amounts of products, which could not be detected under the experimental conditions.

### FPS and crtE gene mutations and over-expression in *X. dendrorhous*

To study the involvement of the *FPS* and *crtE* genes in the astaxanthin and ergosterol biosynthetic pathways of *X. dendrorhous, FPS* and *crtE* deletion and over-expressing mutant strains were constructed.

**Figure 3:**
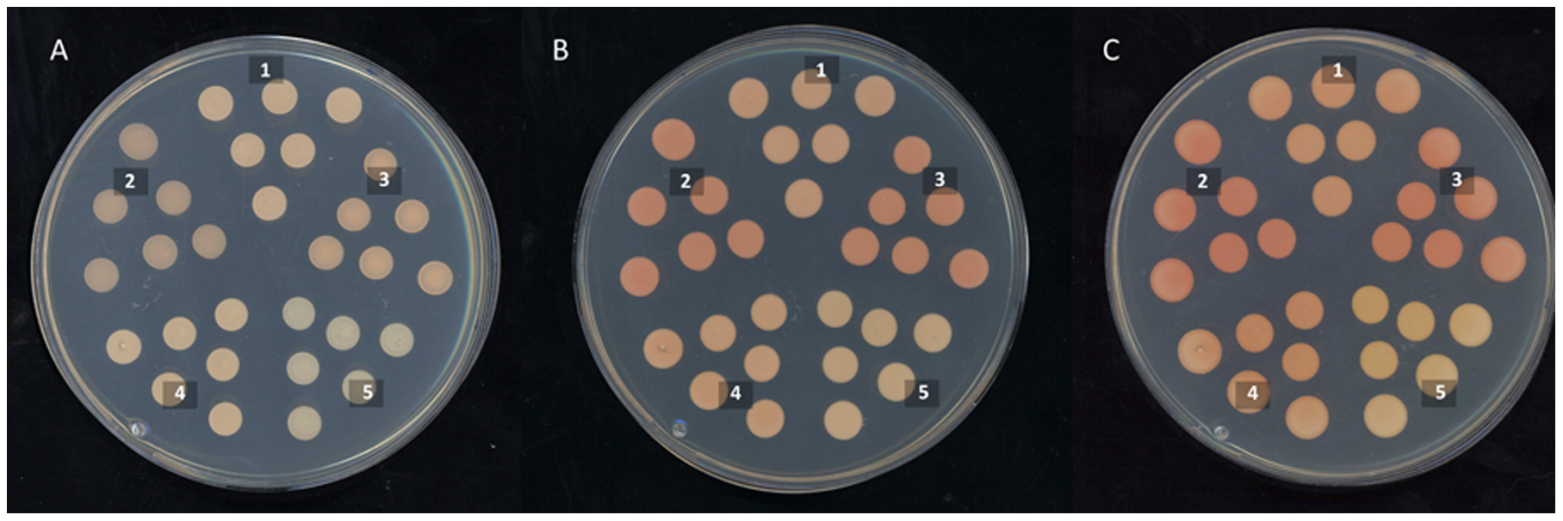
Colony pigmentation of the *X. dendrorhous* wild-type strain and the *FPS* and *crtE* deletion and over-expressing transformants over time. Micro-drops of strains UCD 67–385 wild-type (1), 385-FPS^(+/+, +1)^ (2), 385-crtE^(+/+, +1)^ (3), 385-FPS^(+/−)^ (4) and 385-crtE^(+/−)^ (5), were seeded on YM-agar plates and incubated at 22°C. Pictures were taken after 1 (Panel A), 3 (Panel B) and 5 (Panel C) days of cultivation. Pigmentation is apparent earlier in both of the gene over-expressing strains (2 and 3). The deletion mutant strains (4 and 5) showed similar pigmentation as the wild-type (1) strain after 3 days of cultivation, but at day 5, the 385-crtE^(+/−)^ strain was paler than the wild-type strain.

To generate deletion mutants, the wild-type strain UCD 67–385 was independently transformed using the linearized plasmids pXd-g*crtE*::*hph* and pXd-g*FPS*::*hph*. In this way, the wild-type genes (*FPS* or *crtE*) were replaced by the DNA fragment containing the resistance marker for transformant strain selection, flanked by DNA sequences that allowed homologous recombination between the transforming DNA and the corresponding target *locus*. Several transformants were selected from each transformation event, and the modified genotype was confirmed by PCR reactions using comprehensive sets of primers (Table S1). Figure S4 shows the genotype analysis of one transformant of each kind, indicating that they are heterozygotes for the *FPS* or the *crtE* genes because they have a mutant allele and still have a wild-type allele. For this reason, these transformants were named 385-FPS^(+/−)^ and 385-crtE^(+/−)^, respectively. This result was expected because the experimental evidence suggests that the UCD 67–385 strain is diploid [42]. Although several attempts at transformant homozygotization using the Double Recombinant Method (DRM) [43] were made, it was not possible to obtain *crtE* [17] and *FPS* homozygous mutants, suggesting that these genes are essential and that their homozygote mutant condition could be lethal. Nevertheless, a different color phenotype is apparent to the naked eye between the parental and the heterozygous strains (Fig. 3). This observation suggests that under substrate limiting conditions, the carbon flux may be preferentially directed towards vital functions instead of carotenogenesis, as the growth curves of both heterozygous strains were not significantly altered (Fig. S5). Additionally, the heterozygous condition supports the gene dose effect in the production of carotenoids in *X. dendrorhous*, which has been described in other carotenogenic heterozygous mutants [17], [44].

Similarly, the *FPS* and *crtE* genes were over-expressed in the wild-type strain UCD 67–385 by transforming with linearized plasmids pXdVexp2-c*FPS* and pXdVexp2-c*crtE*; a transformant strain of each type was selected and named 385-FPS^(+/+, +1)^ and 385-crtE^(+/+, +1)^, respectively. In the same way as the heterozygous mutant strains (Fig. S4), PCR-based genotype analyses of the transformant strains confirmed that the over-expression cassettes were integrated at one of the *int locus* alleles. Under visual inspection, the colonies of both over-expressing mutants have a more intense red color than the parental strain (Fig. 3). Additionally, the 385-FPS^(+/+, +1)^ colonies have a glossy appearance, in contrast to the *X. dendrorhous* wild-type colonies, which are usually opaque. The *crtE* over-expression affected the biomass production, as 385-crtE^(+/+, +1)^ cultures reached a lower OD_600_ than did the parental strain (8.76±0.62 versus 12.8±0.93) after 120 h of culture at 22°C in YM media with constant agitation (Fig. S5). This fact suggests that *crtE* over-expression favors the carbon flux towards carotenogenesis at the expense of other metabolic pathways, negatively impacting the strain growth. Conversely, *FPS* over-expression does not affect strain growth, most likely because this condition would favor FPP production, which is also a sterol biosynthetic pathway precursor. Finally, in both over-expressing strains, pigmentation is apparent to the naked eye earlier than in the parental strain and in both heterozygote mutant strains, suggesting that carotenogenesis begins earlier in the *FPS* and *crtE* over-expressing strains (Fig. 3).

### Carotenoids and sterols in *X. dendrorhous* wild-type, FPS and crtE mutant strains

To confirm the visual inspection observations, total carotenoids and sterols were extracted from the wild-type and 385-FPS^(+/−)^, 385-crtE^(+/−)^, 385-FPS^(+/+, +1)^ and 385-crtE^(+/+, +1)^ mutant strains after 72 and 120 h of culture in YM media with constant agitation (Table 5). As expected, the total carotenoid content and/or composition were different in the mutant strains with respect to the wild-type strain. Statistical analysis (Student's t test, p<0.01) confirmed that the *FPS* and *crtE* over-expressing strains have a higher carotenoid content and a slight increase in the astaxanthin fraction compared with the parental strain after 72 h of incubation. Previously, a similar carotenoid content increment (approximately 1.4-fold) was reported by over-expressing the *crtE* gene in *X. dendrorhous* [19]. Conversely, the total carotenoid content in the heterozygote *FPS* and *crtE* mutant strains was not significantly different from the wild-type strain (Student's t test, p<0.01), but the composition was altered, mainly by an increase of beta-carotene, echinenone, OH-echinenone, cantaxanthin and phoenicoxanthin at the expense of the astaxanthin fraction.

**Table 5:** Carotenoid composition in *FPS* and *crtE* mutants and wild-type *X. dendrorhous* strains (in ppm, µg per g of dry yeast weight).

	Strains (72 h of cultivation)				
Carotenoids	UCD 67–385	385-FPS^(+/+, +1)^	385-crtE^(+/+, +1)^	385-FPS^(+/−)^	385-crtE^(+/−)^
**Total**	**376.3±5.52 (100)**	**472.9±25.6 (100)**	**513.4±12.9 (100)**	**380.8±21.4 (100)**	**388.0±5.8 (100)**
Astaxanthin	305.9±12.9 (81.3)	428.4±9.5 (90.6)	451.1±12.5 (87.9)	231.0±32.8 (60.7)	200.4±34.9 (51.6)
Phoenicoxanthin	50.1±4.0 (13.3)	30.0±4.5 (6.3)	38.0±1.9 (7.4)	60.0±2.3 (15.8)	62.5±1.5 (16.1)
Cantaxanthin	9.0±0.6 (2.4)	9.6±2.0 (2.0)	10.5±2.7 (2.0)	15.3±2.7 (4.0)	14.6±4.0 (3.8)
OH-echinenone	4.8±3.0 (1.3)	ND	4.4±4.0 (0.9)	10.5±6.8 (2.8)	25.9±9.7 (6.7)
Echinenone	1.7±1.7 (0.5)	ND	3.9±2.9 (0.8)	28.9±8.9 (7.6)	34.0±5.9 (8.8)
β-carotene	3.1±3.1 (0.8)	ND	ND	38.0±18.7(10.0)	48.0±14.0 (12.4)

	Strains (120 h of cultivation)				
Carotenoids	UCD 67–385	385-FPS^(+/+, +1)^	385-crtE^(+/+, +1)^	385-FPS^(+/−)^	385-crtE^(+/−)^
**Total**	**436.4±11.5(100)**	**514.9±4.1 (100)**	**559.1±30.3 (100)**	**437.5±26.6 (100)**	**440.3±8.82 (100)**
Astaxanthin	366.7±22.2 (84.0)	466.6±17.6 (90.6)	500.4±18.7 (89.5)	237.0±14.6 (54.2)	241.6±15.3 (54.9)
Phoenicoxanthin	45.5±10.8 (10.4)	36.4±10.3 (7.1)	46.1±10.6 (8.2)	80.2±4.1 (18.3)	74.3±0.3 (16.9)
Cantaxanthin	7.2±2.7 (1.6)	4.9±2.6 (1.0)	4.0±2.6 (0.7)	16.3±1.6 (3.7)	13.7±1.4 (3.1)
OH-echinenone	6.0±0.9 (1.4)	0.9±0.0 (0.2)	3.4±0.9 (0.6)	28.3±2.2 (6.5)	39.1±4.2 (8.9)
Echinenone	1.2±0.7 (0.3)	1.7±0.0 (0.3)	1.5±1.5 (0.3)	32.4±4.4 (7.4)	31.0±3.9 (7.0)
β-carotene	5.2±0.1 (1.2)	1.0±0.0 (0.2)	2.1±2.1 (0.4)	41.3±6.7 (9.4)	40.7±6.8 (9.2)

Table shows the mean values ± standard error of the mean of the results of three independent cultures. Percentage relative to total carotenoids is indicated in parentheses.

ND: Not detected.

As mentioned above, the fundamental phenotypic differences between the five strains analyzed in this work can most likely be explained by carbon flux distribution. Thus, the *FPS* and *crtE* over-expressing strains produce more of the carotenogenic pathway precursors, such as FPP and GGPP, while their production should be reduced in the heterozygous mutants. In *X. dendrorhous*, carotenogenesis is induced when glucose in the medium is depleted [25], [45], [46], which occurs close to the stationary phase of growth. Thus, carotenogenesis begins when cultures stop growing and the carbon flux is directed towards carotenoid biosynthesis. However, the *FPS* and *crtE* over-expressing strains might provide an excess of substrate, which might favor an early start for carotenogenesis. This result contrasts with the heterozygous *FPS* and *crtE* mutant strains, in which the growth is not altered (Fig. S5) and the limited carotenogenic substrates may be directed towards biomass formation, thus delaying carotenogenesis (Fig. 3).

Conversely, the altered carotenoid composition in the heterozygous *FPS* and *crtE* mutants may support the hypothesis of the formation of a carotenogenic complex in *X. dendrorhous* [47]. Under this hypothesis, a varying level of one of the protein components of the complex might affect the sequence of the reactions, thus affecting the end products that are formed [47]. In this sense, the carotenogenic complex assembly may possibly be driven, at least in part, by the availability of carotenogenic substrates. Then, under limited substrate concentrations (this would be the case of the heterozygous *FPS* and *crtE* mutants), an incomplete carotenogenic complex might be assembled, favoring the formation of intermediary carotenoids other than astaxanthin. This would not be the case in the *FPS* and *crtE* over-expressing strains, in which a higher amount of carotenogenic substrates might favor an earlier assembly of a carotenogenic complex, leading to an earlier and a higher carotenoid production.

Finally, in contrast to what we expected, no significant differences regarding the ergosterol production among the *X. dendrorhous* transformants and the wild-type parental strain were observed. However, after 120 h of cultivation, there was a small reduction of the ergosterol content in the wild-type strain, which was only statistically significant when compared with the strain 385-crtE^(+/+, +1)^ that had an ergosterol content increment (about 36% more).

### FPS and crtE relative transcript levels and prenyl transferase activity in *X. dendrorhous* deletion and over-expressing transformants

**Figure 4:**
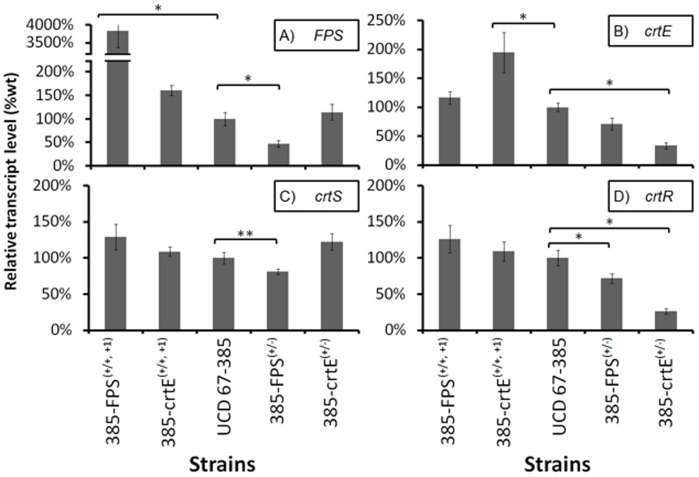
Transcript level changes in the over-expressing and deletion mutant strains versus the parental wild-type strain. The *FPS* (A), *crtE* (B), *crtS* (C) and *crtR* (D) genes expression in transformant and wild-type strains was determined by RT-qPCR and normalized to the actin gene expression after 72 h of cultivation. The respective gene transcript level in the control (wild-type strain) was considered to be 100%. Values are the mean ± standard error of the mean (SEM) of three independent experiments. (*p≤0,01, **p≤0,05; Student's t test).

To assess whether the decrease or increase of the *FPS* and *crtE* gene dose effectively changes mRNA levels, RT-qPCR analyses were performed for wild-type, heterozygous and over-expressing strains grown under the same conditions. Total RNA was extracted after 72 h of culture to determine the relative *FPS* and *crtE* transcript levels, which were normalized to the transcript levels of the actin gene [25]. The RT-qPCR analyses revealed that the relative transcript levels of the *FPS* gene in the 385-FPS^(+/+, +1)^ strain was significantly increased with respect to the wild-type and it was reduced to about half of the wild-type value under the heterozygous condition. However, the relative transcript levels of the *crtE* gene in the 385-crtE^(+/+, +1)^ strain was slightly increased and it was decreased in the 385-crtE^(+/−)^ strain (Fig. 4).

Unlike other astaxanthin-producing organisms, the synthesis of astaxanthin from β-carotene in *X. dendrorhous* is catalyzed by a cytochrome P450 system [48] composed of the cytochrome P450 enzyme (astaxanthin synthase, encoded by the *crtS* gene [49], [50]) and the cytochrome P450 reductase (CPR, encoded by the *crtR* gene [44]). Given that carotenoid composition in both heterozygous mutant strains, 385-FPS^(+/−)^ and 385-crtE^(+/−)^, was different with respect to the wild-type strain, mainly by the reduction of the astaxanthin fraction and an increase of intermediary carotenoids between β-carotene and astaxanthin, the expression of the genes involved at this step, was analyzed (Fig. 4). The relative *crtS* transcript levels showed only a slight reduction in the 385-FPS^(+/−)^ strain. However, the relative *crtR* transcript levels were significantly reduced in both heterozygous strains, while the *FPS* and *crtE* over-expressing strains showed no significant changes. Previous studies have shown that mutation of one of the *crtR* alleles in the diploid UCD 67–385 strain affects the carotenoid composition, decreasing the astaxanthin fraction in favor of β-carotene and other intermediary carotenoids [44]. Thus, our results suggest that the relative *crtR* transcript levels reduction in strains 385-FPS^(+/−)^ and 385-crtE^(+/−)^ could also be responsible for their altered phenotypes. Considering the vast number of reactions in which CPR enzymes are involved, the expression and activity regulation of CPR is generally a complex process that involves several mechanisms [51] that act at different levels. The mechanism by which the *crtE* and *FPS* gene deletions affect the *crtR* transcript levels requires further investigation.

Finally, prenyl transferase activities were determined in soluble protein extracts from the five analyzed *X. dendrorhous* strains, which were obtained after 72 h of cultivation under the conditions described in Materials and Methods (Table 4). Prenyl transferase activity, mainly FPP-synthase, was increased by 85% in the *FPS* over-expressing strain while prenyl transferase activity, measured with FPP, remained unchanged compared to the wild-type strain UCD 67-385. Conversely, the prenyl transferase activity with FPP was increased in 144% in the *crtE* over-expressing mutant, while the activity measured with DMAPP remained unchanged. However, C_20_^14^C-products are a minor fraction in all cases. Also, C_10_ and C_15_^14^C-products can be formed in the presence of FPP as a consequence of IPP isomerase activity that achieves the isomerization of IPP to DMAPP. In enzymatic assays with UCD 67–385 protein extracts and only including IPP as substrate, C_10_, C_15_ and C_20_ were obtained, which evidences IPP isomerase activity in these extracts. However, this endogenous IPP isomerase activity would only affect the enzymatic assays including FPP as substrate, since no DMAPP is added to the assay. On the other hand, the enzymatic assays including DMAPP as substrate, should not be affected as substrates were used at saturated concentrations, so the IPP isomerase activity would be insignificant.

The prenyl transferase activity in heterozygous strain extracts using DMAPP or FPP as a substrate showed no significant differences compared to the wild-type strain (Table 4). Probably, the methodology that we used might not be sensitive enough to detect variations in the prenyl transferase activity in the protein extracts from the strains that we assayed. Also, a transcript level reduction, which we observed for the respective gene in the heterozygous strains, does not necessarily lead to a reduction of the enzymatic activity with the same magnitude.

The results reported here demonstrate that in *X. dendrorhous*, two prenyl transferases act sequentially to synthesize GGPP from DMAPP and IPP precursors: an FPP-synthase (encoded by *FPS*) that produces FPP and an GGPP-synthase (encoded by *crtE*) that transforms the C_15_ prenyl pyrophosphate into GGPP. The accumulation of ^14^C-FPP (detected as C_15_ alcohol in the allylic fraction) in the above assays indicates that the reaction catalyzed by the *crtE* gene product would be rate-limiting in the biosynthetic pathway, which thus represents a possible target for astaxanthin production improvement. Considering that the DNA assembler methodology, which allows integration in the genome of several DNA fragments during one transformation event, was proven to be effective and successful in *X. dendrorhous* [52], *crtE* could be simultaneously over-expressed together with other rate-limiting astaxanthin production genes to favor the synthesis of carotenoids in *X. dendrorhous*.

## Conclusions

In *X. dendrorhous*, GGPP is synthesized from IPP and DMAPP by two prenyl transferase enzymes that act sequentially. In the first stage, an FPP-synthase (encoded by the *FPS* gene) produces FPP in two steps: GPP is first produced by the condensation of IPP and DMAPP, and the addition of a second IPP molecule to GPP then produces FPP. In the second stage, a GGPP-synthase (encoded by the *crtE* gene) produces GGPP by adding a third molecule of IPP to FPP. Both of the genes described in this work, *FPS* and *crtE*, represent good target candidates to enhance the astaxanthin production in *X. dendrorhous* by metabolic engineering approaches.

## Supporting Information

Figure S1 **Plasmids constructed in this work.** In each plasmid illustration, the relevant features such as endonuclease recognition sites and primer binding sites (thin arrows) are shown. Some elements of the original plasmid (pBluescript SK-) were kept in the figure and are shown in gray. The pBluescript SK- skeleton was kept as light gray. Plasmids: A) pXd-g*FPS* harbors a 2.5 kb DNA fragment carrying the *X. dendrorhous FPS* gene. Green, thick arrows represent the nine exons of the *FPS* gene. B) pXd-g*FPS*::*hph* was constructed from pXd-g*FPS*, which was digested with *Eco*RV and *Bgl*II to release a 2.0 kb DNA fragment that contained the *FPS* gene that was replaced by a hygromycin B resistance cassette (HygR, represented by an ochre, thick arrow). For transformation purposes, pXd-g*FPS*::*hph* was linearized with *Ava*I. C) pXd-g*crtE* harbors a 3.3 kb DNA fragment carrying the *X. dendrorhous crtE* gene. Light blue, thick arrows represent the nine exons of the *crtE* gene. D) pXd-g*crtE*::*hph* was constructed from pXd-g*crtE*, which was digested with *Eco*RV to release a 2.0 kb DNA fragment that contained the *crtE* gene that was replaced by a hygromycin B resistance cassette (HygR, represented by an ochre, thick arrow). For transformation purposes, pXd-g*crtE*::*hph* was linearized with *Kpn*I and *Sma*I. E) The pXdVexp2 expression vector was constructed by cloning an *X. dendrorhous* non-coding genomic region (Int, represented by a light orange, thick arrow) to target the integration into the *X. dendrorhous* genome. The Int region was interrupted by inserting the *X. dendrorhous* ubiquitin promoter (Ubi-P, represented by a light purple, thick arrow) and the GPD terminator (GPDT, represented by a dark purple, thick arrow), with a *Hpa*I site between them to insert the gene being expressed. The hygromycin B cassette for transformant selection (HygR, represented by a ochre, thick arrow) was also included. F) The insertion of *FPS* or *crtE* cDNA at the *Hpa*I site of pXdVexp2, yielded pXdVexp2-c*FPS* and pXdVexp2-c*crtE*, respectively.
(TIF)

Figure S2 **Representative chromatogram from prenyl product analysis by TLC.** The prenyl alcohol products that were obtained in the prenyl transferase activity assays were resolved by TLC. The radioactivity detected on each of the TLC-plate fragments is displayed in each graph. The peaks corresponding to the C_10_, C_15_ and C_20_ products are indicated. Graphs correspond to the following enzymatic assay: A) protein extracts from wild-type *X. dendrorhous* strain with DMAPP as additional substrate, B) protein extracts from *E. coli* BL21+FPS strain with DMAPP as additional substrate, C) protein extracts from *E. coli* BL21+crtE strain with FPP as additional substrate and D) a mixture of protein extracts from *E. coli* strains BL21+FPS and BL21+crtE with DMAPP as additional substrate.
(TIF)

Figure S3 **SDS-PAGE analysis of recombinant** ***E. coli*** **protein extracts.** The SDS-PAGE analysis of the protein extracts from induced (+) or not induced (-) recombinant *E. coli* cultures: BL21+pET28 (negative control), BL21+crtE and BL21+FPS is shown. Arrows indicate the recombinant protein band. M: Molecular marker PageRuler™ Thermos (170, 130, 100, 70, 55, 40, 35, 25, 15, 10 kDa).
(TIF)

Figure S4 **PCR-based analysis of the** ***FPS*** **and** ***crtE*** **gene deletion in** ***X. dendrorhous*** **.** PCR analyses to confirm the insertion of the Hygromycin B resistance cassette and the replacement of the target gene of the parental wild-type UCD 67–385 strain (wt). The resulting heterozygous strains (+/−) are 385-FPS^(+/−)^ (upper gel photographs) and 385-crtE^(+/−)^ (lower gel photographs), including the PCR negative control without DNA (−). Between the upper and lower gel photographs, a scheme is included to represent the primer sets (shown in arrows) that were used, the expected PCR-product size and the target DNA. The primers used in the analyses depended on the gene deletion that was studied (*crtE* or *FPS* gene) and were: 1: FPSnewF or crtE_CDS_F1, 2: FPSnewR or crtE_CDS_R1, 3: FPS_Out_F7 or crtE_Out_F1, 4: TEF Antisense, 5: GPDT Sec F and 6: FPS_Out_R1 or crtER2 (Table S1). The scheme shading is in accordance with Figure S1. M: Molecular marker: lambda DNA digested with *Hin*dIII (23.1, 9.4, 6.6, 4.4, 2.3, 2.0 and 0.6 kb).
(TIF)

Figure S5 **Growth curves of the** ***X. dendrorhous*** **strains analyzed in this work.** The yeast strains were grown in YM rich medium at 22°C with constant agitation. Growth curve values correspond to the mean ± standard error of the mean from three independent cultures.
(TIF)

Table S1 **Primers designed and used in this work.**
(DOCX)

## References

[1] Misawa N (2011) Pathway engineering for functional isoprenoids. Current Opinion in Biotechnology 22: 627–633. 21310602 10.1016/j.copbio.2011.01.002

[2] Sacchettini JC, Poulter CD (1997) Creating isoprenoid diversity. Science 277: 1788–1789. 9324768 10.1126/science.277.5333.1788

[3] Liang P, Ko T, Wang AH (2002) Structure, mechanism and function of prenyltransferases. European Journal of Biochemistry 269: 3339–3354. 12135472 10.1046/j.1432-1033.2002.03014.x

[4] Lange BM, Rujan T, Martin W, Croteau R (2000) Isoprenoid biosynthesis: the evolution of two ancient and distinct pathways across genomes. Proc Natl Acad Sci U S A 97: 13172–13177. 11078528 10.1073/pnas.240454797 PMC27197

[5] Lee P, Schmidt-Dannert C (2002) Metabolic engineering towards biotechnological production of carotenoids in microorganisms. Applied Microbiology and Biotechnology 60: 1–11. 12382037 10.1007/s00253-002-1101-x

[6] Lichtenthaler HK (2000) Non-mevalonate isoprenoid biosynthesis: enzymes, genes and inhibitors. Biochemical Society Transactions 28: 785–789. 11171208

[7] Ohnuma S-, Suzuki M, Nishino T (1994) Archaebacterial ether-linked lipid biosynthetic gene. Expression cloning, sequencing, and characterization of geranylgeranyl-diphosphate synthase. Journal of Biological Chemistry 269: 14792–14797. 8182085

[8] Caplin BE, Hettich LA, Marshall MS (1994) Substrate characterization of the *Saccharomyces cerevisiae* protein farnesyltransferase and type-I protein geranylgeranyltransferase. Biochimica et Biophysica Acta (BBA)-Protein Structure and Molecular Enzymology 1205: 39–48. 8142482 10.1016/0167-4838(94)90089-2

[9] Saito K, Fujisaki S, Nishino T (2007) Short-chain prenyl diphosphate synthase that condenses isopentenyl diphosphate with dimethylallyl diphosphate in ispA null *Escherichia coli* strain lacking farnesyl diphosphate synthase. Journal of Bioscience and Bioengineering 103: 575–577. 17630132 10.1263/jbb.103.575

[10] Takaichi S (2011) Carotenoids in algae: distributions, biosyntheses and functions. Marine Drugs 9: 1101–1118. 21747749 10.3390/md9061101 PMC3131562

[11] Goodwin TW (1984) The Biochemistry of Carotenoids, vol. II. Animals, Chapman Hall, London

[12] Gaziano JM, Hennekens CH (1993) The Role of Beta-Carotene in the Prevention of Cardiovascular Disease. Annals of the New York Academy of Sciences 691: 148–155. 8129284 10.1111/j.1749-6632.1993.tb26166.x

[13] Schiedt K, Leuenberger FJ, Vecchi M, Glinz E (1985) Absorption, retention and metabolic transformations of carotenoids in rainbow trout, salmon and chicken. Pure and Applied Chemistry 57: 685–692.

[14] Guerin M, Huntley ME, Olaizola M (2003) *Haematococcus* astaxanthin: applications for human health and nutrition. Trends in Biotechnology 21: 210–216. 12727382 10.1016/S0167-7799(03)00078-7

[15] Andrewes AG, Phaff HJ, Starr MP (1976) Carotenoids of *Phaffia rhodozyma*, a red-pigmented fermenting yeast. Phytochemistry 15: 1003–1007.

[16] Kajiwara S, Fraser PD, Kondo K, Misawa N (1997) Expression of an exogenous isopentenyl diphosphate isomerase gene enhances isoprenoid biosynthesis in *Escherichia coli* . Biochemical Journal 324: 421. 9182699 10.1042/bj3240421 PMC1218447

[17] Niklitschek M, Alcaíno J, Barahona S, Sepúlveda D, Lozano C, et al (2008) Genomic organization of the structural genes controlling the astaxanthin biosynthesis pathway of *Xanthophyllomyces dendrorhous* . Biological Research 41: 93–108. 18769767 10.4067/S0716-97602008000100011

[18] Visser H, Sandmann G, Verdoes JC (2005) Xanthophylls in fungi. editors. Microbial Processes and Products. Springer. pp. 257–272.

[19] Breitenbach J, Visser H, Verdoes JC, van Ooyen AJJ, Sandmann G (2011) Engineering of geranylgeranyl pyrophosphate synthase levels and physiological conditions for enhanced carotenoid and astaxanthin synthesis in *Xanthophyllomyces dendrorhous* . Biotechnology Letters 33: 755–761. 21165672 10.1007/s10529-010-0495-2

[20] Sambrook J, Russell DW (2001) Molecular cloning: a laboratory manual. Volume 1–3..

[21] Cifuentes V, Hermosilla G, Martínez C, León R, Pincheira G, et al (1997) Genetics and electrophoretic karyotyping of wild-type and astaxanthin mutant strains of *Phaffia rhodozyma* . Antonie van Leeuwenhoek 72: 111–117. 9298189 10.1023/a:1000200119447

[22] Chomczynski P, Sacchi N (1987) Single-step method of RNA isolation by acid guanidinium thiocyanate-phenol-chloroform extraction. Analytical Biochemistry 162: 156–159. 2440339 10.1006/abio.1987.9999

[23] Lodato P, Alcaino J, Barahona S, Retamales P, Jimenez A, et al. (2004) Study of the expression of carotenoid biosynthesis genes in wild-type and deregulated strains of *Xanthophyllomyces dendrorhous* (Ex.: *Phaffia rhodozyma*). Biological Research 83–94. 10.4067/s0716-97602004000100009 15174308

[24] Boyle JS, Lew AM (1995) An inexpensive alternative to glassmilk for DNA purification. Trends in Genetics 11: 8. 7900196 10.1016/s0168-9525(00)88977-5

[25] Lodato P, Alcaíno J, Barahona S, Niklitschek M, Carmona M, et al (2007) Expression of the carotenoid biosynthesis genes in *Xanthophyllomyces dendrorhous* . Biological Research 40: 73. 17657357 10.4067/s0716-97602007000100008

[26] Livak KJ, Schmittgen TD (2001) Analysis of Relative Gene Expression Data Using Real-Time Quantitative PCR and the 2− ΔΔCT Method. Methods 25: 402–408. 11846609 10.1006/meth.2001.1262

[27] Kanehisa M, Goto S (2000) KEGG: kyoto encyclopedia of genes and genomes. Nucleic Acids Research 28: 27–30. 10592173 10.1093/nar/28.1.27 PMC102409

[28] Płochocka D, Karst F, Świeżewska E, Szkopińska A (2000) The role of *ERG20* gene (encoding yeast farnesyl diphosphate synthase) mutation in long dolichol formation. Molecular modeling of FPP synthase. Biochimie 82: 733–738. 11018290 10.1016/s0300-9084(00)01155-x

[29] Jiang Y, Proteau P, Poulter D, Ferro-Novick S (1995) *BTS1* encodes a geranylgeranyl diphosphate synthase in *Saccharomyces cerevisiae* . Journal of Biological Chemistry 270: 21793–21799. 7665600 10.1074/jbc.270.37.21793

[30] Hoshino TO, K; Setoguchi Y (1999) DNA sequences encoding enzymes involved in production of isoprenoids. F. HOFFMANN-LA ROCHE AG European Patent Application: EP 0 955 363 A2:

[31] Adrio JL, Veiga M (1995) Transformation of the astaxanthin-producing yeast *Phaffia rhodozyma* . Biotechnology Techniques 9: 509–512. 10.1007/BF00311214 7586031

[32] Loto I, Gutiérrez MS, Barahona S, Sepúlveda D, Martínez-Moya P, et al (2012) Enhancement of carotenoid production by disrupting the C22-sterol desaturase gene (*CYP61*) in *Xanthophyllomyces dendrorhous* . BMC Microbiology 12: 235. 23075035 10.1186/1471-2180-12-235 PMC3552872

[33] Fell JW, Blatt GM (1999) Separation of strains of the yeasts *Xanthophyllomyces dendrorhous* and *Phaffia rhodozyma* based on rDNA IGS and ITS sequence analysis. Journal of Industrial Microbiology and Biotechnology 23: 677–681. 10455500 10.1038/sj.jim.2900681

[34] An G-H, Schuman DB, Johnson EA (1989) Isolation of *Phaffia rhodozyma* mutants with increased astaxanthin content. Applied and Environmental Microbiology 55: 116–124. 16347815 10.1128/aem.55.1.116-124.1989 PMC184064

[35] Shang F, Wen S, Wang X, Tan T (2006) Effect of nitrogen limitation on the ergosterol production by fed-batch culture of *Saccharomyces cerevisiae* . Journal of Biotechnology 122: 285–292. 16488499 10.1016/j.jbiotec.2005.11.020

[36] Mekkrienkrai D, Sando T, Hirooka K, Sakdapipanich J, Tanaka Y, et al (2004) Cloning and characterization of farnesyl diphosphate synthase from the rubber-producing mushroom *Lactarius chrysorrheus* . Bioscience, Biotechnology and Biochemistry 68: 2360–2368. 10.1271/bbb.68.2360 15564677

[37] Koike-Takeshita A, Koyama T, Obata S, Ogura K (1995) Molecular cloning and nucleotide sequences of the genes for two essential proteins constituting a novel enzyme system for heptaprenyl diphosphate synthesis. Journal of Biological Chemistry 270: 18396–18400. 7629164 10.1074/jbc.270.31.18396

[38] Cantera JJL, Kawasaki H, Seki T (2002) Farnesyl diphosphate synthase gene of three phototrophic bacteria and its use as a phylogenetic marker. International Journal of Systematic and Evolutionary Microbiology 52: 1953–1960. 12508853 10.1099/00207713-52-6-1953

[39] Ohnuma Si HK, Ohto C, Nishino T (1997) Conversion from Archaeal Geranylgeranyl Diphosphate Synthase to Farnesyl Diphosphate Synthase. Two amino acids before the first aspartate-rich motif solely determine eukaryotic farnesyl diphosphate synthase activity. Journal of Biological Chemistry 272: 5192–5198. 9030588 10.1074/jbc.272.8.5192

[40] Sitthithaworn W, Kojima N, Viroonchatapan E, Suh D-Y, Iwanami N, et al (2001) Geranylgeranyl diphosphate synthase from *Scoparia dulcis* and *Croton sublyratus*. Plastid localization and conversion to a farnesyl diphosphate synthase by mutagenesis. Chemical and Pharmaceutical Bulletin 49: 197–202. 11217109 10.1248/cpb.49.197

[41] Gao Y, Honzatko RB, Peters RJ (2012) Terpenoid synthase structures: a so far incomplete view of complex catalysis. Natural Product Reports 29: 1153–1175. 22907771 10.1039/c2np20059g PMC3448952

[42] Hermosilla G, Martínez C, Retamales P, León R, Cifuentes V (2003) Genetic determination of ploidy level in *Xanthophyllomyces dendrorhous* . Antonie van Leeuwenhoek 84: 279–287. 14574105 10.1023/a:1026090008405

[43] Niklitschek M, Baeza M, Fernández-Lobato M, Cifuentes V (2012) Generation of astaxanthin mutants in *Xanthophyllomyces dendrorhous* using a Double Recombination Method based on Hygromycin Resistance. editors. Microbial Carotenoids From Fungi. Springer. pp. 219–234. 10.1007/978-1-61779-918-1_15 22711129

[44] Alcaíno J, Barahona S, Carmona M, Lozano C, Marcoleta A, et al (2008) Cloning of the cytochrome p450 reductase (*crtR*) gene and its involvement in the astaxanthin biosynthesis of *Xanthophyllomyces dendrorhous* . BMC Microbiology 8: 169. 18837978 10.1186/1471-2180-8-169 PMC2575211

[45] Marcoleta A, Niklitschek M, Wozniak A, Lozano C, Alcaíno J, et al (2011) Glucose and ethanol-dependent transcriptional regulation of the astaxanthin biosynthesis pathway in *Xanthophyllomyces dendrorhous* . BMC Microbiology 11: 1–11. 21861883 10.1186/1471-2180-11-190 PMC3184065

[46] Wozniak A, Lozano C, Barahona S, Niklitschek M, Marcoleta A, et al (2011) Differential carotenoid production and gene expression in *Xanthophyllomyces dendrorhous* grown in a nonfermentable carbon source. FEMS Yeast Research 11: 252–262. 21205159 10.1111/j.1567-1364.2010.00711.x

[47] Verdoes JC, Sandmann G, Visser H, Diaz M, van Mossel M, et al (2003) Metabolic engineering of the carotenoid biosynthetic pathway in the yeast *Xanthophyllomyces dendrorhous* (*Phaffia rhodozyma*). Applied and Environmental Microbiology 69: 3728–3738. 12839738 10.1128/AEM.69.7.3728-3738.2003 PMC165150

[48] Alcai´no J, Fuentealba M, Cabrera R, Baeza M, Cifuentes V (2012) Modeling the Interfacial Interactions between CrtS and CrtR from *Xanthophyllomyces dendrorhous*, a P450 System Involved in Astaxanthin Production. Journal of Agricultural and Food Chemistry 60: 8640–8647. 22897793 10.1021/jf302287f

[49] Álvarez V, Rodríguez-Sáiz M, de la Fuente JL, Gudiña EJ, Godio RP, et al (2006) The *crtS* gene of *Xanthophyllomyces dendrorhous* encodes a novel cytochrome-P450 hydroxylase involved in the conversion of β-carotene into astaxanthin and other xanthophylls. Fungal Genetics and Biology 43: 261–272. 16455271 10.1016/j.fgb.2005.12.004

[50] Ojima K, Breitenbach J, Visser H, Setoguchi Y, Tabata K, et al (2006) Cloning of the astaxanthin synthase gene from *Xanthophyllomyces dendrorhous* (*Phaffia rhodozyma*) and its assignment as a β-carotene 3-hydroxylase/4-ketolase. Molecular Genetics and Genomics 275: 148–158. 16416328 10.1007/s00438-005-0072-x

[51] van den Brink JM, Punt PJ, van Gorcom RFM, van Den Hondel CAMJJ (2000) Regulation of expression of the *Aspergillus niger* benzoate para-hydroxylase cytochrome P450 system. Molecular and General Genetics 263: 601–609. 10852481 10.1007/s004380051207

[52] Contreras G, Barahona S, Rojas MC, Baeza M, Cifuentes V, et al (2013) Increase in the astaxanthin synthase gene (*crtS*) dose by *in vivo* DNA fragment assembly in *Xanthophyllomyces dendrorhous* . BMC Biotechnology 13: 84. 24103677 10.1186/1472-6750-13-84 PMC3852557

