# Supplementary figures and images for: Functional Characterization of the Xanthophyllomyces dendrorhous Farnesyl Pyrophosphate Synthase and Geranylgeranyl Pyrophosphate Synthase Encoding Genes That Are Involved in the Synthesis of Isoprenoid Precursors
Source: PLoS One. 2014 May 5;9(5):e96626. doi: 10.1371/journal.pone.0096626 (PMC4010515; doi:10.1371/journal.pone.0096626)

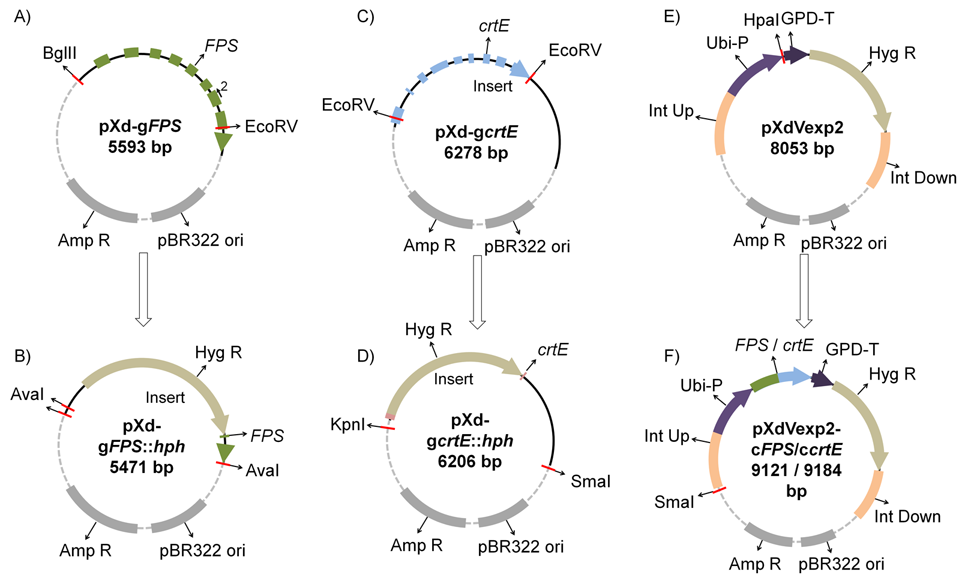

Supplement: Figure S1 — Plasmids constructed in this work. In each plasmid illustration, the relevant features such as endonuclease recognition sites and primer binding sites (thin arrows) are shown. Some elements of the original plasmid (pBluescript SK-) were kept in the figure and are shown in gray. The pBluescript SK- skeleton was kept as light gray. Plasmids: A) pXd-gFPS harbors a 2.5 kb DNA fragment carrying the X. dendrorhous FPS gene. Green, thick arrows represent the nine exons of the FPS gene. B) pXd-gFPS::hph was constructed from pXd-gFPS, which was digested with EcoRV and BglII to release a 2.0 kb DNA fragment that contained the FPS gene that was replaced by a hygromycin B resistance cassette (HygR, represented by an ochre, thick arrow). For transformation purposes, pXd-gFPS::hph was linearized with AvaI. C) pXd-gcrtE harbors a 3.3 kb DNA fragment carrying the X. dendrorhous crtE gene. Light blue, thick arrows represent the nine exons of the crtE gene. D) pXd-gcrtE::hph was constructed from pXd-gcrtE, which was digested with EcoRV to release a 2.0 kb DNA fragment that contained the crtE gene that was replaced by a hygromycin B resistance cassette (HygR, represented by an ochre, thick arrow). For transformation purposes, pXd-gcrtE::hph was linearized with KpnI and SmaI. E) The pXdVexp2 expression vector was constructed by cloning an X. dendrorhous non-coding genomic region (Int, represented by a light orange, thick arrow) to target the integration into the X. dendrorhous genome. The Int region was interrupted by inserting the X. dendrorhous ubiquitin promoter (Ubi-P, represented by a light purple, thick arrow) and the GPD terminator (GPDT, represented by a dark purple, thick arrow), with a HpaI site between them to insert the gene being expressed. The hygromycin B cassette for transformant selection (HygR, represented by a ochre, thick arrow) was also included. F) The insertion of FPS or crtE cDNA at the HpaI site of pXdVexp2, yielded pXdVexp2-cFPS and pXdVexp2-ccrtE, respectivel [file pone.0096626.s001.tif]

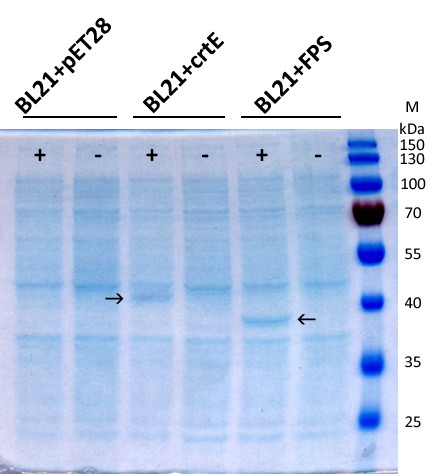

Supplement: Figure S2 — Representative chromatogram from prenyl product analysis by TLC. The prenyl alcohol products that were obtained in the prenyl transferase activity assays were resolved by TLC. The radioactivity detected on each of the TLC-plate fragments is displayed in each graph. The peaks corresponding to the C10, C15 and C20 products are indicated. Graphs correspond to the following enzymatic assay: A) protein extracts from wild-type X. dendrorhous strain with DMAPP as additional substrate, B) protein extracts from E. coli BL21+FPS strain with DMAPP as additional substrate, C) protein extracts from E. coli BL21+crtE strain with FPP as additional substrate and D) a mixture of protein extracts from E. coli strains BL21+FPS and BL21+crtE with DMAPP as additional substrate. (TIF) [file pone.0096626.s002.tif]

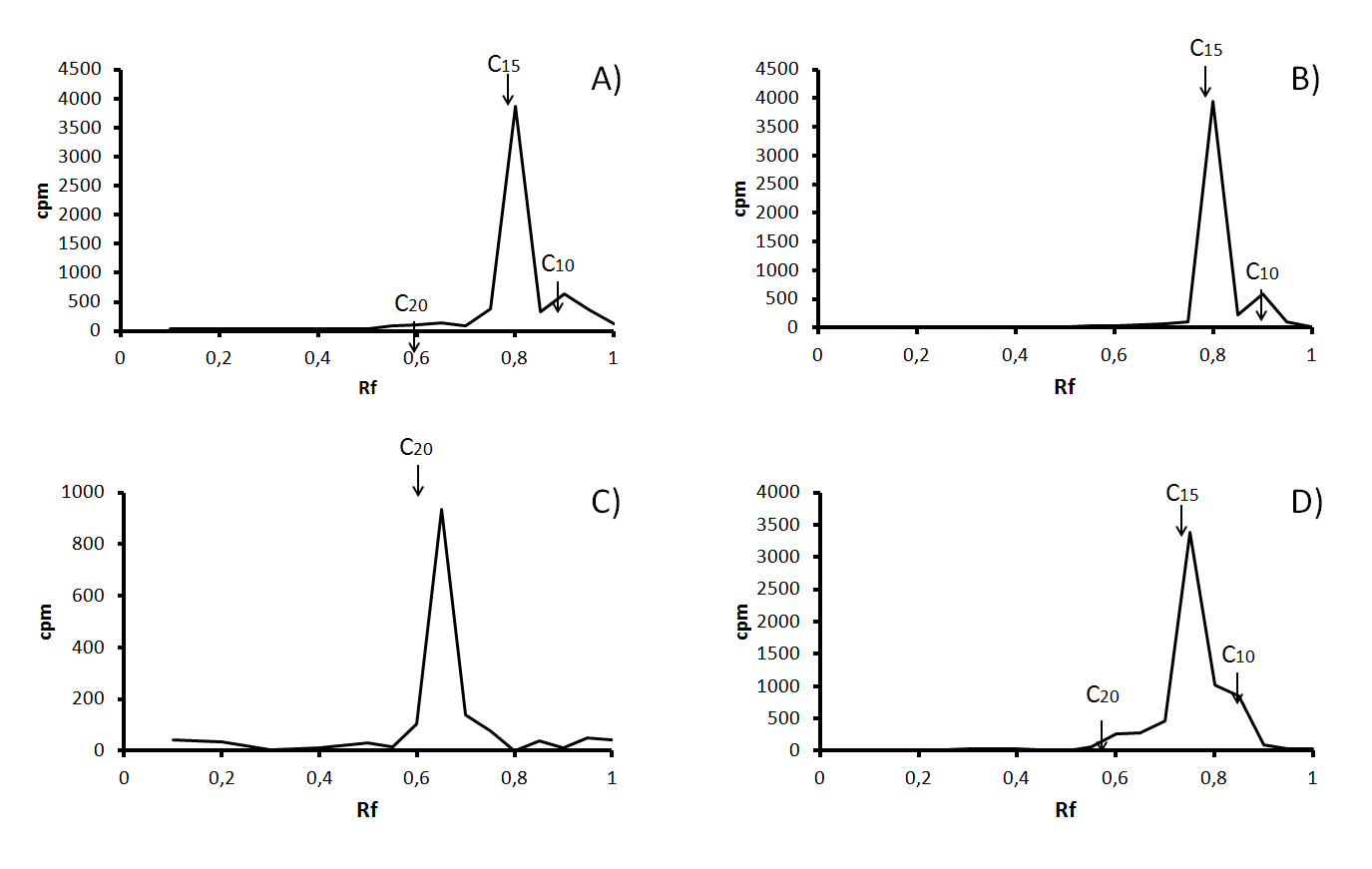

Supplement: Figure S3 — SDS-PAGE analysis of recombinant E. coli protein extracts. The SDS-PAGE analysis of the protein extracts from induced (+) or not induced (-) recombinant E. coli cultures: BL21+pET28 (negative control), BL21+crtE and BL21+FPS is shown. Arrows indicate the recombinant protein band. M: Molecular marker PageRuler™ Thermos (170, 130, 100, 70, 55, 40, 35, 25, 15, 10 kDa). (TIF) [file pone.0096626.s003.tif]

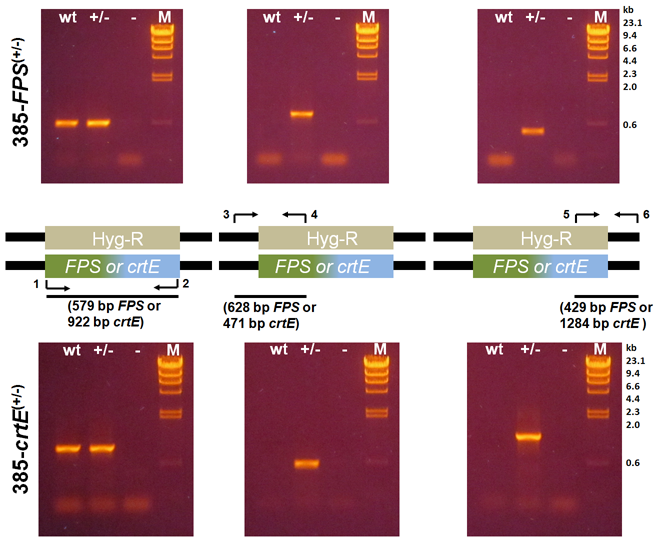

Supplement: Figure S4 — PCR-based analysis of the FPS and crtE gene deletion in X. dendrorhous . PCR analyses to confirm the insertion of the Hygromycin B resistance cassette and the replacement of the target gene of the parental wild-type UCD 67–385 strain (wt). The resulting heterozygous strains (+/−) are 385-FPS(+/−) (upper gel photographs) and 385-crtE(+/−) (lower gel photographs), including the PCR negative control without DNA (−). Between the upper and lower gel photographs, a scheme is included to represent the primer sets (shown in arrows) that were used, the expected PCR-product size and the target DNA. The primers used in the analyses depended on the gene deletion that was studied (crtE or FPS gene) and were: 1: FPSnewF or crtE_CDS_F1, 2: FPSnewR or crtE_CDS_R1, 3: FPS_Out_F7 or crtE_Out_F1, 4: TEF Antisense, 5: GPDT Sec F and 6: FPS_Out_R1 or crtER2 (Table S1). The scheme shading is in accordance with Figure S1. M: Molecular marker: lambda DNA digested with HindIII (23.1, 9.4, 6.6, 4.4, 2.3, 2.0 and 0.6 kb). (TIF) [file pone.0096626.s004.tif]

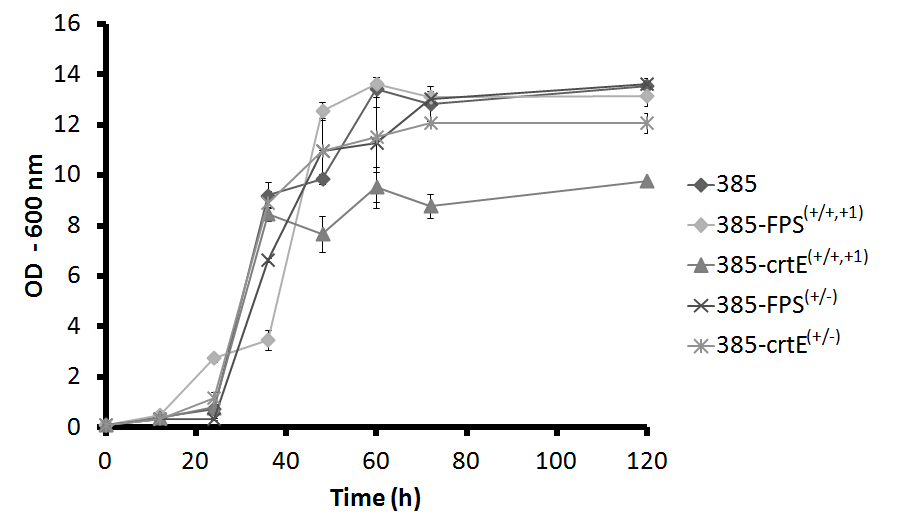

Supplement: Figure S5 — Growth curves of the X. dendrorhous strains analyzed in this work. The yeast strains were grown in YM rich medium at 22°C with constant agitation. Growth curve values correspond to the mean ± standard error of the mean from three independent cultures. (TIF) [file pone.0096626.s005.tif]
