# Supplementary material for: Functional Characterization of the Xanthophyllomyces dendrorhous Farnesyl Pyrophosphate Synthase and Geranylgeranyl Pyrophosphate Synthase Encoding Genes That Are Involved in the Synthesis of Isoprenoid Precursors
Source: PLoS One. 2014 May 5;9(5):e96626. doi: 10.1371/journal.pone.0096626 (PMC4010515; doi:10.1371/journal.pone.0096626)
Supplement: Table S1 — Primers designed and used in this work. (DOCX) [file pone.0096626.s006.docx]

**Table S1: Primers designed and used in this work**

| **Primer** | **Sequence 5’ to 3’** | **Target and Special Features** |
| --- | --- | --- |
| GPDT Sec F | ttcctatcgccttcatcctc | *hph* cassette |
| TEF Antisense | ctattcctttgccctcggac | *hph* cassette |
| mActF-RT | ccgccctcgtgattgataac | *Actin* gene |
| mActR-RT | tcaccaacgtaggagtcctt | *Actin* gene |
| Int_Pre_Fw | acccgctcccgatccgattt | Non coding insertion sequence |
| Int_Post_Rv | agttcggactcgagtgtgatc | Non coding insertion sequence |
| mcrtE-NdeI-R | cccgaa**catatg**tcacagagggatatcggc | *crtE* gene, reverse primer. The *Nde*I site is underlined |
| mcrtE-NcoI-F2 | gactag**ccatgg**attacgcgaacatcctc | *crtE* gene, forward primer. The *Nco*I site is underlined |
| mcrtEF | atggattacgcgaacatcctc | *crtE* gene |
| mcrtER | tcacagagggatatcggctag | *crtE* gene |
| crtE_Out_F1 | atgactcttgccggcgatttga | upstream *crtE* gene |
| crtER2 | ctgaccaaagcacaatcgttc | downstream *crtE* gene |
| mcrtEF-RT | tgttggcatgctacataccg | *crtE* gene cDNA (for qPCR) |
| mcrtER-RT | gttgggcgaagcttgaagat | *crtE* gene cDNA (for qPCR) |
| crtE_CDS_F1 | atcccatctctgtgtgtgttcc | *crtE* gene |
| crtE_CDS_R1 | ccttctgtgaggtcctctgcaa | *crtE* gene |
| mFPS-NdeI-R | cccgaa**catatg**ttacttgcttcgcttatagac | *FPS* gene, reverse primer. The *Nde*I site is underlined |
| mFPS-NcoI-F | gactag**ccatgg**ccactacgcctgaag | *FPS* gene, forward primer. The *Nco*I site is underlined |
| mFPSF2-RT | tggtacaaagttgagggagtgtc | *FPS* gene cDNA (for qPCR) |
| mFPSR2-RT | agcggtcaacagatcgatgag | *FPS* gene cDNA (for qPCR) |
| FPS_Out_F7 | caagctagctcttcgtctgt | upstream *FPS* gene |
| FPS_Out_R1 | tgaccctcccttctcttcct | downstream *FPS* gene |
| FPSnewF | ctatgtcgatctgctagagc | *FPS* gene |
| FPSnewR | tcgagcacatcatcctgaac | *FPS* gene |
| FPSF | catcgccattaacgacgc | *FPS* gene |
| FPSR | ccaggagcacttgttgtc | *FPS* gene |
| FPSORF1F | atgttcaagtgtcaggcga | *FPS* gene |
| FPSORF2F | atgtccactacgcctgaaga | *FPS* gene 5' ORF end |
| FPSORF1R | ttacttgcttcgcttatagacc | *FPS* gene 3' ORF end |
| mcrtSF-RT | atggctcttgcagggtttga | *crtS* gene cDNA (for qPCR) |
| mcrtSR-RT | tgctccataagctcgatcccaa | *crtS* gene cDNA (for qPCR) |
| mcrtRF-RT | ctgggaaacaagacctacga | *crtR* gene cDNA (for qPCR) |
| mcrtRR-RT | ggaacctcggttacgacaaa | *crtR* gene cDNA (for qPCR) |
